# ICG Clearance in Assessing Cirrhotic Patients with Hepatocellular Carcinoma for Major Hepatic Resection

**DOI:** 10.1155/1997/69231

**Published:** 1997

**Authors:** Seigo Kitano, Yang-II Kim

**Affiliations:** 1 Department of Surgery I Oita Medical University Oita 879-55 Japan; 2 Department of Surgery Catholic University of Taegu-Hyosung School of Medicine Taegu 705-034 Korea

## Abstract

*Objective*: To deWne the safety of major hepatectomy for hepatocellular carcinoma (HCC) associated with cirrhosis and the selection criteria for surgery in terms of hospital mortality.

*Design*: Major hepatectomy for HCC in the presence of cirrhosis is considered to be contraindicated by many surgeons because the reported mortality rate is high (26% to 50%). Previous workers recommended that only selected patients with Child's A status or indocyanine green (ICG) retention at 15 minutes of less than 10% undergo major hepatectomy. A survery was made, therefore, of our patients with HCC and cirrhosis undergoing major hepatectomy between 1989 and 1994.

*Setting*: A tertiary referral center.

*Patients*: The preoperative, intraoperative, and post-operative data of 54 patients with cirrhosis who had major hepatectomy were compared with those of 25 patients with underlying chronic active hepatitis and 22 patients with normal livers undergoing major hepatectomy for HCC. The data had been prospectively collected.

*Intervention*: Major hepatectomy, defined as resection of two or more liver segments by Goldsmith and Woodburn nomenclature, was performed on all the patients. Main Outcome Measure: Hospital mortality, which was defined as death within the same hospital admission for the hepatectomy.

*Results*: Preoperative liver function in patients with cirrhosis was worse than in those with normal livers. The intraoperative blood loss was also higher (P=.01), but for patients with cirrhosis, chronic active hepatitis, and normal livers, the hospital mortality rates (13%, 16%, and 14%, respectively) were similar. The hospital mortality rate for patients with cirrhosis in the last 2 years of the study was only 5%. Patients with cirrhosis could tolerate up to 10 L of blood loss and survive the major hepatectomy. By discriminant analysis, an ICG retention of 14% at 15 minutes was cutoff level that could maximally separate the patients with cirrhosis with and without mortality.

*Conclusion*: Major hepatectomy for HCC in the presence of cirrhosis is associated with a mortality rate that is not different from the rate for patients with normal livers. An ICG retention of 14% at 15 minutes would serve as a better selection criterion than the 10% previously used.


### ICG CLEARANCE IN ASSESSING CIRRHOTIC PATIENTS WITH HEPATOCELLULAR CARCINOMA FOR MAJOR HEPATIC RESECTION

### ABSTRACT

*Fan, S-T., Lai, E.C.S., Lo, C-M., Ng, I.O.L. and Wong, J. (1995) Hospital mortality of major hepatectomy for hepatocellular carcinoma associated with cirrhosis. Archives of Surgery; 130: 198–203*

***Objective:*** **To deWne the safety of major hepatectomy for hepatocellular carcinoma (HCC) associated with cirrhosis and the selection criteria for surgery in terms of hospital mortality.**
***Design:*** **Major hepatectomy for HCC in the presence of cirrhosis is considered to be contraindicated by many surgeons because the reported mortality rate is high (26% to 50%). Previous workers recommended that only selected patients with Child's A status or indocyanine green (ICG) retention at 15 minutes of less than 10% undergo major hepatectomy. A survery was made, therefore, of our patients with HCC and cirrhosis undergoing major hepatectomy between 1989 and 1994.**
***Setting:*** **A tertiary referral center.**
***Patients:*** **The preoperative, intraoperative, and post-operative data of 54 patients with cirrhosis who had major hepatectomy were compared with those of 25 patients with underlying chronic active hepatitis and 22 patients with normal livers undergoing major hepatectomy for HCC. The data had been prospectively collected.**
***Intervention:*** **Major hepatectomy, defined as resection of two or more liver segments by Goldsmith and Woodburn nomenclature, was performed on all the patients.**
**Main Outcome Measure: Hospital mortality, which was defined as death within the same hospital admission for the hepatectomy.**
***Results:*** **Preoperative liver function in patients with cirrhosis was worse than in those with normal livers. The intraoperative blood loss was also higher (P=.01), but for patients with cirrhosis, chronic active hepatitis, and normal livers, the hospital mortality rates (13%, 16%, and 14%, respectively) were similar. The hospital mortality rate for patients with cirrhosis in the last 2 years of the study was only 5%. Patients with cirrhosis could tolerate up to 10 L of blood loss and survive the major hepatectomy. By discriminant analysis, an ICG retention of 14% at 15 minutes was cutoff level that could maximally separate the patients with cirrhosis with and without mortality.**
***Conclusion:*** **Major hepatectomy for HCC in the presence of cirrhosis is associated with a mortality rate that is not different from the rate for patients with normal livers. An ICG retention of 14% at 15 minutes would serve as a better selection criterion than the 10% previously used.**
**(Arch Surg. 1995; 130: 198–203)**

KEY WORDS: Hepatocellular carcinoma, cirrhosis, liver resection

### PAPER DISCUSSION

Liver resection can now be carried out in many major centres in order to treat hepatic diseases; the operative mortality is less than 5%, if the associated liver parenchyma is normal[1]. The existence of cirrhosis has been traditionally considered a contraindication, particularly for extensive hepatectomy because mortality and morbidity rates are unacceptably high[2]. Cirrhotic patients have metabolic, circulatory and coagulation problems linked to the diminished capacity of the diseased liver[3,4].

In 54 cirrhotic patients treated by major liver resection. Dr Fan and his group confirmed data on some patients, but raised the question of safety criteria for extensive liver resection. Major hepatectomy was defined as resection of two or more of hepatic segments, by the classification of U.S.A. workers (Goldsmith and Woodburne). Though the majority of their cirrhotic patients (89%, 48/54) underwent liver resection of more than the right lobe, the hospital mortality was 13%, a value compatible with data on patients with chronic active hepatitis and normal livers (16% and 14%, respectively). By discriminant analysis, and ICG retention of 14% at 15 minutes (ICG R15) determined before the surgery was the cut off level that could maximally discriminate the presence or absence of hospital mortality. The authors propose that this preoperative value is a better risk parameter to predict posthepatectomy liver failure than the previously used 10%.

Risk factors for post-hepatectomy liver failure include massive bleeding, extensive resection, ischemia and postoperative infection, particularly for patients with chronic liver diseases. With respect to intraoperative haemorrhage in the present study, the non-surviving cirrhotic patients had a substantial blood loss (11.0±2.5L, Mean±SE) of three times more than that for the survivors (3.3±0.4L, $p<0.02$). Thus, massive haemorrhage during hepatectomy can lead to hepatic failure and a fatal outcome. However, the hemostatics must be considered for this group, because even under normal conditions of the associated liver parenchyma there is a 1600ml of blood loss requiring blood replacement[5]. As a consequence, the majority of such patients (86%, 19/22) are maintained on mechanical ventilation following resection of the "normal" liver.

It is generally considered that there is no correlation between conventional liver function tests and post-hepatectomy mortality. Assessing functional reserve of the liver is difficult, and methods range from a relatively simple classification system to more complex ones[6]. The ICG test, as a simple parameter, is considered to reflect the degree of hepatic dysfunction more accurately[7]. The present report provides guidelines for a safer, major lobectomy in cirrhotic patients. However, the extent of the remnant liver parenchyma can be significantly greater even under the same conditions of lobectomy, because of a compensatory hypertrophy due to the huge tumor to be resected. In this setting, since the neoplastic tissue occupying the lobe to be resected does not contain functioning hepatocytes, resection of a large tumor even by lobectomy does not unduly affect the functional volume. In other words, ablation ofmainly the non-functioning liver mass by the major hepatectomy should not worsen the preoperative total liver function, for instance, the preoperative ICG value. When considering the relatively large mass lesions in the present series, the authors could preoperatively estimate the post-resectional functional volume of individual livers. We emphasize that surgeons need to know the critical functional reserve to sustain life after tumor resection. For this purpose, Okamoto *et al*[8]. Advocate a combination use of ICG clearance test and liver volumetry. Other investigators add a loading test (maximal removal rate of ICG, R max) to quantify the degree of hepatic functional reserve which is very difficult to evaluate using the standard dye excretion rate[9]. Further information is awaited regarding the preoperative estimation of functional remnant volume in hepatic surgery.

Dr Fan and colleagues have provided information which requires extended study on a large series of major-hepatectomized cirrhotics. Risk factors using the ICG clearance test in liver surgery should be given due attention.

### REFERENCES

1. Iwatsuki, S. and Startzl, T.E. (1988) Personal experience with 411 hepatic resections. *Annals of Surgery* **208**, 421–34.
2. Franco, D., Capussotti, L. and Smadja, C. *et al.* (1990) Resection of hepatocellular carcinomas: Results in 72 European patients with cirrhosis. *Gastroenterology*, **98**, 733–38
3. Siegel, J.H., Greenspan, M., Cohn, J.D. and Del Guercio, L.R.M. (1968) The prognostic implications of altered physiology in operations in operations for portal hypertension. *Surgery, Gynecology & Obstetrics*, **126**, 249–62.
4. Lin, T.Y. and Chen, C.C. (1965) Metabolic function and regeneration of cirrhotic and noncirrhotic livers after hepatic lobectomy in man. *Annals of Surgery*, **162**, 959–72.
5. Doci, R., Gennari, L. and Bignami, P. *et al.* (1995) Morbidity and mortality after hepatic resection of metastases from colorectal cancer. *British Journal of Surgery*, **82**, 377–81.
6. MacIntosh, E. L. and Minuk, G. Y. (1992) Hepatic resection in patients with cirrhosis and hepatocellular carcinoma. *Surgery, Gynecology & Obstetrics*, **174**, 245–54.
7. Kaplowitz, N., Eberle, D., and Yamada, T. (1982) Hepatology-a textbook of liver disease, Philadelphia: W.B. Saunders Co.
8. Okamoto, E., Kyo, A., Yamanaka, N., Tanaka, N. and Kumata, K. (1984) Prediction of the safe limits of hepatectomy by combined volumetric and functional measurements in patients with impaired hepatic function. *Surgery*, **95**, 586–92.
9. Mizumoto, R., Kawarada, Y. and Noguchi, T. (1979) Preoperative estimation of operative risk in liver surgery, with special reference to functional reserve of the remnant liver following major hepatic resection. *Japanese Journal of Surgery*, **9**, 343–49.

Seigo. Kitano
Department of Surgery I
Oita Medical University
Oita 879-55, Japan

Yang-Il Kim
Department of Surgery
Catholic University of Taegu-Hyosung
School of Medicine
Taegu 705-034, Korea

